# Developmental control of noradrenergic system by SLITRK1 and its implications in the pathophysiology of neuropsychiatric disorders

**DOI:** 10.3389/fnmol.2022.1080739

**Published:** 2023-01-04

**Authors:** Minoru Hatayama, Jun Aruga

**Affiliations:** Department of Medical Pharmacology, Nagasaki University Graduate School of Biomedical Sciences, Nagasaki, Japan

**Keywords:** SLITRK1, noradrenaline, monoamines, obsessive - compulsive disorder, animal disease model, synaptic adhesion molecule

## Abstract

SLITRK1 is a neuronal transmembrane protein with neurite development-and synaptic formation-controlling abilities. Several rare variants of SLITRK1 have been identified and implicated in the pathogenesis of Tourette’s syndrome, trichotillomania, and obsessive–compulsive disorder, which can be collectively referred to as obsessive–compulsive-spectrum disorders. Recent studies have reported a possible association between bipolar disorder and schizophrenia, including a revertant of modern human-specific amino acid residues. Although the mechanisms underlying SLITRK1-associated neuropsychiatric disorders are yet to be fully clarified, rodent studies may provide some noteworthy clues. Slitrk1-deficient mice show neonatal dysregulation of the noradrenergic system, and later, anxiety-like behaviors that can be attenuated by an alpha 2 noradrenergic receptor agonist. The noradrenergic abnormality is characterized by the excessive growth of noradrenergic fibers and increased noradrenaline content in the medial prefrontal cortex, concomitant with enlarged serotonergic varicosities. Slitrk1 has both cell-autonomous and cell-non-autonomous functions in controlling noradrenergic fiber development, and partly alters Sema3a-mediated neurite control. These findings suggest that transiently enhanced noradrenergic signaling during the neonatal stage could cause neuroplasticity associated with neuropsychiatric disorders. Studies adopting noradrenergic signal perturbation *via* pharmacological or genetic means support this hypothesis. Thus, Slitrk1 is a potential candidate genetic linkage between the neonatal noradrenergic signaling and the pathophysiology of neuropsychiatric disorders involving anxiety-like or depression-like behaviors.

## 1. Introduction

The mammalian Slitrk family of proteins consists of six transmembrane proteins with two leucine-rich repeat domains (Slitrk1, Slitrk2, Slitrk3, Slitrk4, Slitrk5, Slitrk6; Aruga and Mikoshiba, 2003; Aruga et al., 2003). They are predominantly and differentially expressed in both immature and mature neural tissues in humans and mice (Aruga et al., 2003; Aruga and Mikoshiba, 2003; Beaubien and Cloutier, 2009; Stillman et al., 2009). They possess the ability to control neurite development (Aruga and Mikoshiba, 2003; Abelson et al., 2005; Katayama et al., 2009) and enhance synapse formation (Takahashi et al., 2012; Tekin et al., 2013; Yim et al., 2013; Beaubien et al., 2016; Kang et al., 2016; Schroeder et al., 2018; Biselli et al., 2021; El Chehadeh et al., 2022; Hatayama et al., 2022). However, these two abilities differ qualitatively and quantitatively among the six members (Aruga and Mikoshiba, 2003; Takahashi et al., 2012; Yim et al., 2013). In this review article, we focus on SLITRK1 and its involvement in the brain functions of health and disease, primarily in view of review articles dealing with the Slitrk family (Proenca et al., 2011; Ko, 2012; Won et al., 2019).

## 2. Variations of human SLITRK1 and their significances

**Figure 1:**
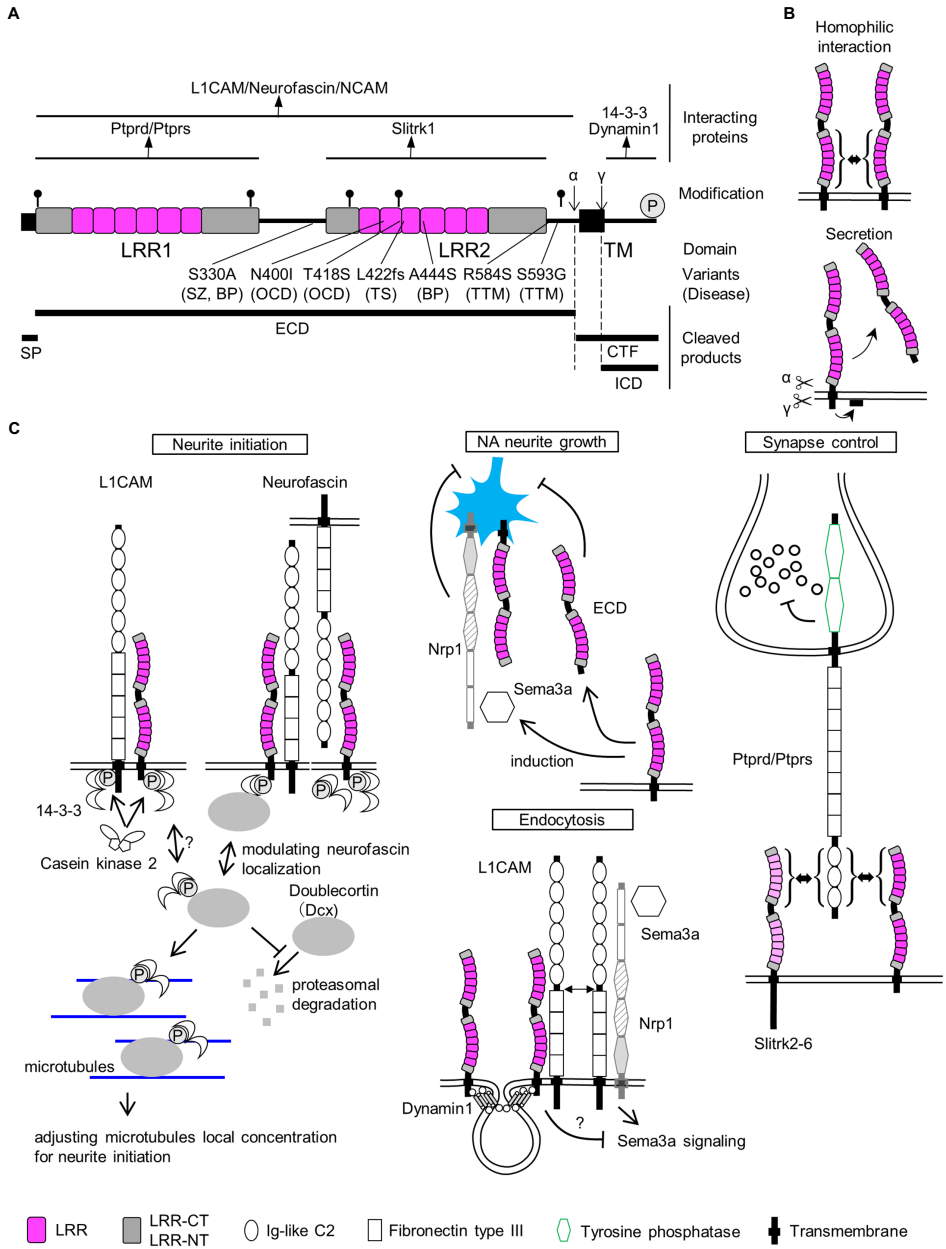
SLITRK1 properties. **(A)** SLITRK1 domain structure, modifications, and binding proteins. LRR, leucine rich repeat; TM, transmembrane. The top lines indicate the regions for the above binding partners. Putative N-glycosylation sites are indicated as *pins* and cleavage sites of secretases are indicated as *α* and *γ,* respectively. The phosphorylation site is indicated as *circled P*. Variants, changes in amino acid residues of SLITRK1 protein; (Continued)FIGURE 1 (Continued)*Disease*, derived disease (SZ, schizophrenia; BP, bipolar disorder; OCD, obsessive–compulsive disorders; TS, Tourette’s syndrome; TTM, trichotillomania). Bottom-thick lines indicate cleaved products. SP, signal peptide; ECD, extra-cellular domain; CTF, C-terminal fragment; ICD, intra-cellular domain. **(B)** *Homophilic interaction*. SLITRK1 forms a homophilic dimer in contact with LRR2 domains. *Secretion*. SLITRK1 is cleaved by α and γ secretases. ECD is secreted while ICD is attached to the cell membrane. **(C)** SLITRK1 molecular functions including the binding partners for each context.

Among the human SLITRK genes, SLITRK1 was the first to be identified as a candidate genetic factor for neuropsychiatric disorders. In 2005, Abelson et al. identified a frameshift mutation (SLITRK1 L422fs) in an individual with Tourette’s syndrome (TS; Figure 1A; Table 1) and in a cohort of TS patients, inv.(13; q31.1; q33.1) and variants in the 3′ untranslated region (SLITRK1 var321) were enriched (Abelson et al., 2005). TS is diagnosed by the sustained presence of both vocal and motor tics, and represent the more severe end of the spectrum of tic disorders (Fernandez et al., 2018). Following the initial report, numerous subsequent studies surveyed SLITRK1 variants in independent TS patient groups. Many studies failed to identify the L422fs or var321 (Deng et al., 2006; Keen-Kim et al., 2006; Verkerk et al., 2006; Chou et al., 2007; Fabbrini et al., 2007; Orth et al., 2007; Pasquini et al., 2008; Scharf et al., 2008; Zimprich et al., 2008; Yasmeen et al., 2013). However, some studies have supported the association between SLITRK1 and TS (Miranda et al., 2009; Karagiannidis et al., 2012; Inai et al., 2015; Alexander et al., 2016). Overall, these results indicate the involvement of SLITRK1 in TS etiology in a small fraction of patients.

**Table 1:** SLITRK1 variants identified in the patients.

Variants		ID	Disease	Sorting	Misfolding	Neurite	Synapse	Conservation	References
S330A	c.988 T > G	rs145628951	SZ, BP	–	–	+	+	Hs	b, e
N400I	c.1199 T > A	−	OCD	+	+	+	+	v	c, d
T418S	c.1252A > T	rs150504822	OCD	+	+	+	+	v	c, d
L422fs	c.1264C > del	rs193302861	TS	+	+	+	N. A.	n/a	a, e
A444S	c.1330G > T	rs1450785142	BP	–	–	+	+	m	e
R584K	c.1751G > A	rs1035448844	TTM	–	–	N. A.	–	m	b, d, e
S593G	c.1177A ≥ G	rs1368546312	TTM	–	–	N. A.	–	m	d, e

Variants, changes in amino acid residues of SLITRK1 protein and nucleotide sequence in SLITRK1 open reading frame; ID, research SNP database ID; Disease, derived disease (SZ, schizophrenia; BP, bipolar disorder; OCD, obsessive–compulsive disorders; TS, Tourette’s syndrome; TTM, trichotillomania); Sorting, effects of mutations in subcellular protein localization (–, comparable to WT protein; +, abnormal distribution); Misfolding, protein misfolding (–, comparable to WT protein; +, abnormal), Neurite, neurite growth controlling ability (+, abnormal; N.A., not available); Synapse, synapse inducing ability (–, comparable to WT; +, defective; N.A., not available); Conservation, extent of evolutionary conservation of the mutated residue (Hs, Homo sapiens; v, vertebrates; m, mammals; n/a, not applicable), References (a, Abelson et al., 2005; b, Zuchner et al., 2006; c, Ozomaro et al., 2013; d, Kang et al., 2016; e, Hatayama et al., 2022).

Conversely, SLITRK1 missense mutations have been reported in trichotillomania (R584K, S593G; Zuchner et al., 2006) and obsessive–compulsive disorders (OCD; N400I, T418S; Figure 1A; Table 1; Ozomaro et al., 2013). Trichotillomania is a chronic behavioral disorder characterized by the recurrent pulling of one’s own hair, leading to hair loss (Grant and Chamberlain, 2016). Obsessive–compulsive disorder is characterized by the presence of obsessions and/or compulsions (Stein et al., 2019). Obsessions are repetitive and persistent thoughts, images, impulses, or urges that are intrusive and unwanted, and are commonly associated with anxiety. Compulsions are repetitive behaviors or mental acts that the individual feels driven to perform in response to an obsession according to rigid rules, or to achieve a sense of “completeness” (Stein et al., 2019). Family and treatment studies have indicated that TS, trichotillomania, and OCD comprise a larger spectrum of conditions (obsessive–compulsive spectrum disorder or obsessive–compulsive and related disorders, hereafter OCRD; Ferrao et al., 2009; Leckman et al., 2010; Stein et al., 2019). In pharmacotherapy for OCRD, monoaminergic neurotransmission is targeted. TS responds to haloperidol (a dopamine D2 receptor antagonist) and clonidine (an adrenergic α2 receptor agonist; Fernandez et al., 2018). Selective serotonin reuptake inhibitors (SSRI) and clomipramines (serotonin–noradrenaline [NA] reuptake inhibitors) are used as first-line drugs for treating OCD (Stein et al., 2019). Clomipramine has some benefits in trichotillomania treatment as well (Hoffman et al., 2021).

In addition to OCRD, a functionally damaging mutation (A444S) was significantly enriched in a bipolar disorder (BPD) patient cohort (Figure 1A; Table 1; Hatayama et al., 2022). Furthermore, a genome-wide association study identified a single nucleotide polymorphism *ca* 780 kb upstream of SLITRK1, associated with schizophrenia (Bansal et al., 2018). Taken together, SLITRK1 appears to be a risk factor for OCRD and other neuropsychiatric diseases. Summarizing the SLITRK1 mutations that have been implicated in neuropsychiatric disorders, missense mutations were clustered in the second leucine-rich repeat domains of this protein (Figure 1A). This may indicate the pathological significance of the second LRR domain.

However, many missense mutations have been identified in SLITRK1 from not only disease cases, but also control subjects upon whole exome sequencing studies for bipolar disorder, schizophrenia, and epilepsy^1^. This indicates that functional variations of SLITRK1 exist even in *healthy* individuals as well. In agreement with this idea, the S330A mutation (5 × 10^−5^ – 3 × 10^−3^ for both cases and controls) affected SLITRK1 function (Figure 1A; Table 1; Hatayama et al., 2022). S330A is a revertant of the A330S mutation that was acquired in SLITRK1 during the evolution of *Homo sapiens* from the common ancestor of *Homo neanderthalensis* (Table 1).

## 3. Basic properties of SLITRK1

### 3.1. Expression in rodent and primate brains

**Figure 2:**
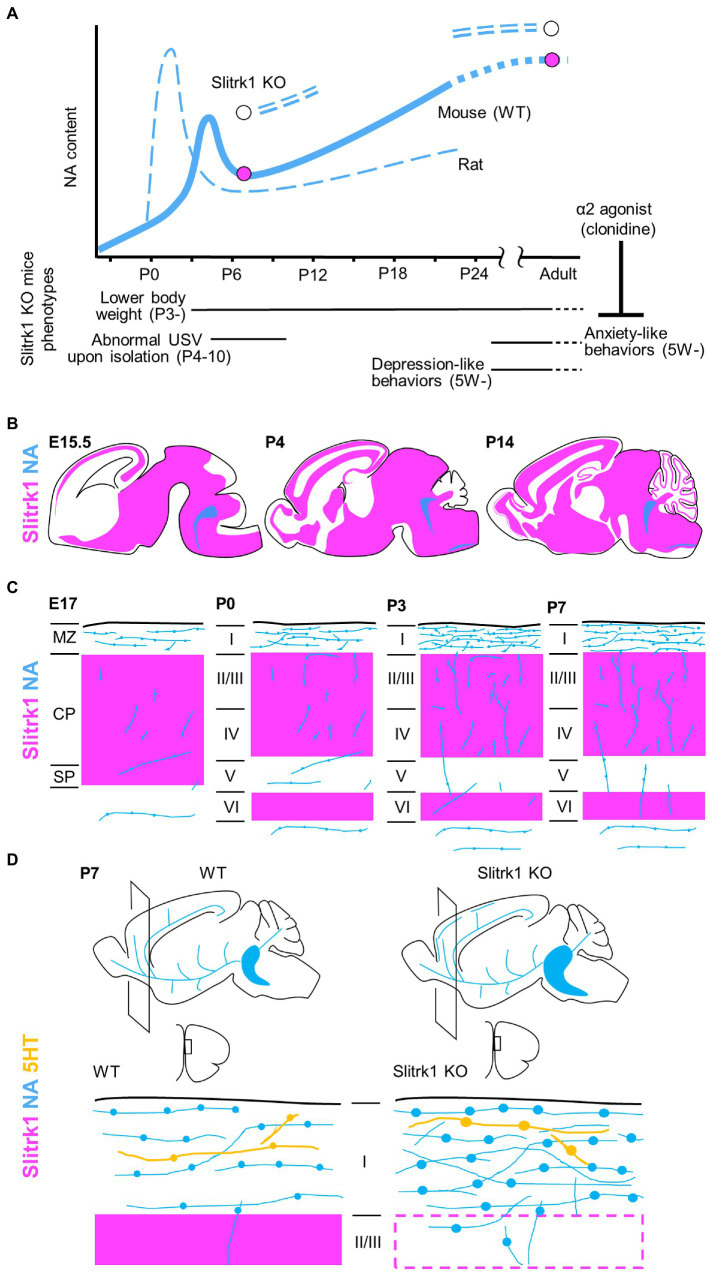
Slitrk1 and NA signalling. **(A)** NA contents in cerebral cortex in developing rodents. The graphs for rat and wild-type (WT) mouse derived from Levitt and Moore (1979). NA contents are increased in PFC of Slitrk1 KO mice both at P7 and adult stages. Slitrk1 KO neurodevelopmental (Continued)FIGURE 2 (Continued)phenotypes are indicated below. Anxiety-like behaviour at adult stage was rescued by α2 agonist clonidine. **(B)** Slitrk1 mRNA distribution (*purple area*) in developing mouse brain from embryonic day (*E*) 15.5, Postnatal (*P*) 4, and P14. *Blue* indicates LC area deduced from DBH (dopamine beta-hydroxylase) expression. Illustrations derived from the Allen brain atlas (https://portal.brain-map.org/). **(C)** Cortical noradrenergic fiber (*blue*) development and Slitrk1 mRNA expression (*purple*). MZ, marginal zone; CP, cortical plate; SP, subplate; I-VI, cortical layers. **(D)** Monoaminergic neuronal phenotypes of Slitrk1 KO mice; the medial prefrontal cortex (mPFC) at P7. Noradrenergic (*blue*), and serotonergic (*orange*) fibers and their varicosities are illustrated. Slitrk1 mRNA distribution (*purple*) is overlapped.

Slitrk1 mRNA is predominantly detected in the brains of both humans and mice (Aruga et al., 2003; Aruga and Mikoshiba, 2003; Fagerberg et al., 2014). In developing mouse brains, at embryonic day (E) 17–18, Slitrk1 mRNA was widely detected in the cerebral cortex, hippocampus, glomerular layer and mitral cell layer of the olfactory bulb, pyramidal cell layer of the hippocampus, striatum, amygdala, septum, thalamus, hypothalamus, superior and inferior colliculus, Purkinje cell layer, deep nuclei of the cerebellum, spinal cord, dorsal root ganglia, trigeminal ganglia, ganglionic cell layer, and inner nuclear layer of the retina (Figure 2B; Aruga and Mikoshiba, 2003; Beaubien and Cloutier, 2009; Stillman et al., 2009). At the adolescent stage (postnatal day (P) 20–30), the highest expression levels were observed in excitatory neuron subtypes (hippocampal CA1, TEGLU21, 0.770; cerebral cortex, TEGLU5, 0.540; midbrain, MEGLU6, 0.763; hindbrain, HBGLU1, 0.501, HBGLU4, 0.572), and afferent nuclei of cranial nerves (V-XII, 0.681; III–V, 0.520), while modest expression was seen in monoaminergic neuron cell types (NA, HBNOR, 0.131; serotonin, HBSER1-4, 0.138–0.362; dopamine, MBDOP2, 0.143)^2^ (Zeisel et al., 2018). In the process of corticogenesis, the expression was first observed at the cortical plate and subplate, and later (P2–) at layers II–IV and VI (Figure 2C; Beaubien and Cloutier, 2009; Stillman et al., 2009; Allen Brain map^3^).

Phylogenetically, SLITRK1 and other SLITRK family proteins exist in vertebrates; however, no orthologs have been identified in invertebrates, including cephalochordates and urochordates. In zebrafish embryos, Slitrk1 expression was detected in the thalamus, hypothalamus, tegmentum, medulla oblongata, and retina (Round et al., 2014). Evolutionary conserved expression profiles have been reported between mouse and chicken (mantle layer and motor neurons of the developing spinal cord; Aruga and Mikoshiba, 2003) and between mouse and zebrafish (ganglionic cell layer and inner nuclear layer of the developing retina; Beaubien and Cloutier, 2009; Round et al., 2014).

### 3.2. Molecular properties of the SLITRK1 protein

In mouse and rat brains, Slitrk1 proteins can be detected in the frontal cortex, hippocampus, amygdala, and olfactory bulb regions (Katayama et al., 2010; Yim et al., 2013). It was abundantly recovered in the synaptosome and postsynaptic density fractions from adult rat brains (Yim et al., 2013). The N-terminal 15 amino acids are predicted to be cleaved^4^ and thought to act as a signal peptide sequence (Figure 1A). Slitrk1 proteins contain six candidate N-glycosylation sites (Figure 1A) and are N-glycosylated (Kajiwara et al., 2009; Yim et al., 2013). Glycosylated forms were detected as 85 kDa and 95 kDa bands whereas that of the non-glycosylated form was 76 kDa (Kajiwara et al., 2009) which matches the calculated molecular weight (76.0 kDa) of SLITRK1 without a signal peptide sequence. The α-secretase-mediated cleavage generates the extracellular domain (ECD), which can be detected as a *ca.* 90 kDa band in the soluble fraction of P3 rat brain lysate, and a carboxy-terminal fragment (CTF, 9.3 kDa; Figures 1A,B; Kajiwara et al., 2009). γ-Secretase cleaves at an intramembranous site, generating an intracellular domain (ICD, 6.5 kDa) that is associated with membrane surfaces (Figures 1A,B; Kajiwara et al., 2009). The secreted SLITRK1 ECD was found to be glycosylated (Figure 1A; Kajiwara et al., 2009). The serine residue near the carboxy terminus (Ser_695_) is phosphorylated by casein kinase II, whereas protein kinase A and C can phosphorylate other sites of the ICD (Figures 1A,C *Neurite initiation*; Kajiwara et al., 2009).

SLITRK1 has shorter ICD (53 aa) than the other SLITRK proteins (SLITRK2-6, 194–298 aa; Aruga et al., 2003). SLITRK1 ICD lacks the conserved carboxy-terminal sequence PDYLXVLE that was similar to the carboxy terminal region of Ntrk neurotrophin receptor proteins (Aruga and Mikoshiba, 2003). Accordingly, the effects on neurite growth in NGF-treated PC12 cells are different between Slitrk1 and Slitrk2-6. Slitrk1 increase single neurite bearing cells without affecting the mean neurite length whereas Slitrk2-6 inhibits nerite outgrowth (Aruga and Mikoshiba, 2003). In terms of synapse inducing abilities, there are no clear contrasting between Slitrk1 and Slitrk2-6 (Figure 1C *Synapse control*; Takahashi et al., 2012; Yim et al., 2013). These results suggest that SLITRK1 ICD possesses unique function among the SLITRK family proteins. Although functional differences among the ECDs of Slitrk family proteins are not clear at this point, whether secreted ECDs exist for Slitrk2-6 awaits further investigation.

### 3.3. Slitrk1-binding proteins

Many Slitrk1-binding proteins have been identified to date (Figure 1A). The LRR1 domain of SLITRK1 ECD physically interacts with receptor-type protein tyrosine phosphatases, PTPRD (Takahashi et al., 2012; Yim et al., 2013), and PTPRS (Yim et al., 2013; Figures 1A,C *Synapse control*). The LRR2 domain of the SLITRK1 ECD is required for homophilic interactions between SLITRK1 proteins (Figures 1A,B; Beaubien et al., 2016) although the significance of the homophilic dimer remains to be clarified. SLITRK1 ECD can also interact with L1 family proteins (Neurofascin, L1CAM, and NCAM; Figures 1A,C *Neurite initiation*, *Endocytosis*; Hatayama et al., 2022). ICD can be bound by seven 14-3-3 family proteins (14-3-3β, YWHAB; 14-3-3γ, YWHAG; 14-3-3ε, YWHAE; 14-3-3η, YWHAH; 14-3-3σ, Sfn, YWHAS; 14-3-3τ, YWHAQ; and 14-3-3ζ, YWHAZ; Kajiwara et al., 2009; Figures 1A,C *Neurite initiation*). The carboxy terminus is predicted to mediate the physical interaction between SLITRK1 and Dynamin1 (Figures 1A,C *Endocytosis*; Hatayama et al., 2022).

## 4. Molecular function of the Slitrk1 protein

### 4.1. Neurite controlling ability

Both overexpression of Slitrk1 and its loss of function can affect neurite patterns (Aruga and Mikoshiba, 2003; Abelson et al., 2005; Kajiwara et al., 2009; Hatayama et al., 2022). In NGF-treated PC12 cells overexpressing Slitrk1, the neuronal population bearing single neurites had increased (Aruga and Mikoshiba, 2003). The overexpression in cortical neurons altered total neurite length in either directions (increased Abelson et al., 2005; Kajiwara et al., 2009; decreased Hatayama et al., 2022) or increased in hippocampal neurons (Kang et al., 2016). The neurite-modulating activities are affected by the carboxy terminal casein kinase II phosphorylation site mutation, S695A (Kajiwara et al., 2009) or BPD-derived mutation (A444S), or the revertant of *Homo sapiens*-specific residue (S330A; Hatayama et al., 2022). The primary cultured locus coeruleus (LC) neurons from Slitrk1-knockout (KO) mice show increased proximal (10–20 μm) neurite numbers but decreased distally (80 μm <; Hatayama et al., 2022). This change in neurite patterning is in line with that observed in Slitrk1-overexpressing PC12 cells (i.e., decreased proximal neurite numbers). Furthermore, addition of SLITRK1 ECD to LC neuron culture increased neurite branch numbers in the proximal region but decreased them in the distal region (Hatayama et al., 2022). The branching pattern similarity between Slitrk1 KO derived neurons and SLITRK1 ECD-treated neurons suggested that secreted Slitrk1 ECD suppresses Slitrk1 function to control neurite development (Figure 1C *NA neurite growth*). Although the role of Slitrk1 ECD is yet to be clarified *in vivo*, it could be involved in the cell non-autonomous regulation of LC nerite growth in developing brains.

To explain neurite controlling, the involvement of the 14-3-3 family proteins has been proposed as the molecular mechanism. Kajiwara et al. showed that a 14-3-3 protein binds to the carboxy-terminus of Slitrk1 in an S695 phosphorylation-dependent manner (Kajiwara et al., 2009). 14-3-3 proteins are known to control neurite initiation (Cornell and Toyo-Oka, 2017). In a hypothetical model of 14-3-3ε-mediated neurite initiation control (Cornell et al., 2016; Cornell and Toyo-Oka, 2017), its binding to doublecortin (Dcx) stabilized Dcx, and 14-3-3/Dcx affected the microtubule dynamics required for neurite formation (Figure 1C *Neurite initiation*).

Dcx and 14-3-3 signaling are associated with not only Slitrk1 but also with its binding partners, L1CAM and Neurofascin. 14-3-3 binds to the phosphorylated serine residue (S1181) in the L1CAM intracellular domain and influenced L1CAM mediated neurite outgrowth (Figure 1C *Neurite initiation*; Ramser et al., 2010). S1181 is phosphorylated by casein kinase II, which gets enhanced by 14-3-3ζ (Figure 1C *Neurite initiation*; Ramser et al., 2010). As for Neurofascin, Dcx physically interacts with Neurofascin and Dcx can modulate the surface distribution of neurofascin in developing cultured rat neurons, where Dcx increases endocytosis of neurofascin from the soma and dendrites (Figure 1C *Neurite initiation*; Yap et al., 2012). As Neurofascin can suppress the effects of Slitrk1 on proximal neurites in both a cis and trans fashion (Figure 1C *Neurite initiation*; Hatayama et al., 2022), Dcx function is predicted to affect the neurite-controlling ability of Slitrk1.

However, considering that 14-3-3 family proteins are multifunctional proteins with more than 200 binding partners (Cornell and Toyo-Oka, 2017), a more accurate picture adapted for Slitrk1-mediated neurite initiation control would be required in the future. For the control of NA fiber growth in the cerebral cortex, a Sema3a and/or Slitrk1 ECD-mediated control mechanism is proposed, as described below.

### 4.2. Synapse controlling ability

Slitrk1 can induce synapses when expressed in both neural and non-neural cells (Takahashi et al., 2012; Yim et al., 2013; Um et al., 2014; Beaubien et al., 2016; Hatayama et al., 2022). In knockdown experiments using hippocampal neurons, excitatory, but not inhibitory synapses are reduced (Yim et al., 2013; Beaubien et al., 2016). Binding to protein tyrosine phosphatases, these results indicate that Slitrk1 induces excitatory synapses through the trans-synaptic interaction with PTPRS, *in vitro*. In the *in vivo* experiments, the short hairpin-mediated knockdown of Slitrk1 in rat CA1 increased the spontaneous excitatory postsynaptic currents frequency and synaptic vesicles at the active zone without affecting dendritic spine density (Schroeder et al., 2018). It was proposed that Slitrk1 exerts its role by selectively recruiting active zone proteins (Schroeder et al., 2018).

The presynapse-organizing protein phosphatase receptors, PTPRD (Takahashi et al., 2012; Yim et al., 2013) and PTPRS (Yim et al., 2013), bind Slitrk1 in a trans-synaptic fashion. PTPRS, but not PTPRD knockdown impaired the artificial synapse formation ability between hippocampal neurons and Slitrk1-expressing HEK293T cells (Yim et al., 2013). The LRR1 domain of Slitrk1 physically interacts with the N-terminal immunoglobulin-like domains in PTPRD, and the trans-interaction subsequently causes clustering of PTPRs (Figure 1C *Synapse control*; Um et al., 2014; Won et al., 2019). It is possible that the clustering of PTPRs mediates the suppression of synaptic vesicles near the active zones.

### 4.3. Endocytosis controlling ability

Slitrk1 can suppress Sema3A (semaphorin3A)-induced endocytosis (Figure 1C *Endocytosis*; Hatayama et al., 2022). The function purportedly involves the binding to Dymamin1 or L1CAM, both of which are known as Slitrk1 binding proteins (Hatayama et al., 2022).

## 5. Behavioral abnormalities in Slitrk1 KO mice

The phenotypes of Slitrk1 KO mice (Katayama et al., 2010; Hatayama et al., 2022) provided clues to consider the role of Slitrk1 at individual levels (Figure 2A). Slitrk1 KO male mice showed lower body weight at as early as P3 during development. The body weight difference stabilizes at one point (compared to control) and again becomes apparent after weaning, to later stages with a 9–14% lower body weight than that of WT mice at the same stage. Female KO mice showed a transiently lower body weight only at P14 (−17%). Isolation-induced ultrasonic vocalization calls were weak at P4 (males) and P7 (both sexes), and the calling rates were lower in females at P7 and P10. Thus, neurodevelopmental phenotypes exist in the neonatal stage in a sex-dependent manner.

Slitrk1 KO mice consistently exhibited decreased locomotor activity, which was manifested at 5 weeks of age (Katayama et al., 2010; Hatayama et al., 2022). The behavioral phenotype at the adult stage involves anxiety-and depression-like phenotypes (Katayama et al., 2010). The anxiety phenotype is characterized by reduced stay time in the open-field apparatus, reduced open-arm stay time, and enhanced freezing responses in fear conditioning tests (Katayama et al., 2010). Depression-like behavior is characterized by increased immobile time in the forced swimming and tail-suspension test (Katayama et al., 2010).

Other behavioral features, such as stereotypy, tremor, seizure, and abnormal repetitive behaviors, were not observed in the timed video recordings of Slitrk1 KO mice (Katayama et al., 2010). None of the responses exhibited abnormalities in the marble burying behavior, Morris water maze, or prepulse inhibition tests (Katayama et al., 2010). Innate reflexes, such as righting, pivoting, rooting, geotaxis, bar holding, grasping, visual place response, auditory startle, and tactile startle, appeared timely during development (Hatayama et al., 2022). Taken together, the neonatal body weight loss, neonatal vocalization abnormalities, and anxiety-like and depression-like behaviors in the adolescent and adult stages feature neurodevelopmental phenotypes in Slitrk1 KO mice (Figure 2A).

## 6. Monoamine disturbance in Slitrk1 KO mice

As many antidepressants and anxiolytics target proteins that control monoamine dynamics, monoamine abundance in Slitrk1 KO mice have been examined (Katayama et al., 2010; Hatayama et al., 2022).

In adult male mice, noradrenaline (NA) and its metabolite, 3-methoxy-4-hydroxyphenylglycol (MHPG), tended to be higher in the three brain regions of the preferential cortex, nucleus accumbens, and stratum in Slitrk1 KO mice. The NA content was significantly higher in the prefrontal cortex (PFC), and the MHPG in the nucleus accumbens, than in wild-type mice (Figure 2A). Further, the serotonin metabolite (5-hydroxyindole acetic acid) content was higher in the nucleus accumbens, and choline content was lower in the Slitrk1 KO striatum (Katayama et al., 2010). Administration of clonidine, an a2-adrenergic agonist that is frequently used to treat patients with Tourette’s syndrome and OCRD, attenuated the anxiety-like behavior of Slitrk1 KO mice (Figure 2A; Katayama et al., 2010), suggesting NA dysregulation to be associated with anxiety-like behaviors.

At the neonatal stage (P7), NA levels were higher in the PFC of male Slitrk1 KO PFC (Figure 2A), but the NA levels were comparable in the female PFC (Hatayama et al., 2022). Furthermore, the levels of the NA metabolite, MHPG decreased in both sexes (Hatayama et al., 2022).

In terms of monoaminergic fiber morphology, NA fiber density was increased two-fold in the P7 PFC of both male and female Slitrk1 KO mice (Figures 2C,D; Hatayama et al., 2022). The size of the LC increased at the same stage (Figure 2D; Hatayama et al., 2022). However, excessive NA innervation in the PFC was limited to the neonatal period and was unclear in the adolescent and adult stages (Hatayama et al., 2022). Therefore, Slitrk1 plays a role in the suppression of noradrenergic projections during the neonatal stage. However, the varicosities in serotonergic fibers are enlarged only in the male Slitrk1 KO PFC (Figure 2D), which is proposed to be due to the actions of excessive NA through the α2-heteroreceptor based on the results of clonidine treatment for neonates (Hatayama et al., 2022).

NA-dynamics are sexually dimorphic (Ngun et al., 2011), and this seems to be associated with the sex differences in vesicular monoamine transporter (VMAT2) function, where female mice possess greater striatal VMAT2 levels/activity (Dluzen and McDermott, 2008), as well as the sex-dependent role of glucocorticoid receptors in the noradrenergic system (Chmielarz et al., 2013). Furthermore, catechol O-methyl transferase (COMT), an NA and dopamine-metabolizing enzyme, exhibits sexual dimorphism (Gogos et al., 1998). Therefore, the role of Slitrk1 in NA fiber suppression is proposed to be primarily responsible for other sexually dimorphic phenotypes (excess NA and serotonergic varicosity enlargement).

## 7. Mechanisms underlying Slitrk1-mediated suppression of neonatal NA fiber

Slitrk1 overexpression in the developing somatosensory cortex reduces NA fibers (Hatayama et al., 2022), supporting the idea that Slitrk1 suppresses NA fiber overgrowth in the neonatal cortex.

When LC cells were cultured from Slitrk1 KO newborn mice, they showed higher and lower neurite complexity, respectively, in the proximal and distal neurites, (Hatayama et al., 2022), indicating the cell-autonomous function of Slitrk1 in controlling LC projections. Conversely, addition of the Slitrk1 ECD domain to the culture medium inhibited neurite development similar to those of Slitrk1 KO-derived neurites (Figure 1C *NA neurite growth*; Hatayama et al., 2022), indicating the non-autonomous function of Slitrk1. The suppression of NA projections by Slitrk1 in the PFC seems valid because Slitrk1 is strongly expressed in the frontal cortex during early postnatal development (Figures 2B,C), and Slitrk1 ECD is produced in the brain by α-secretase-mediated cleavage (Kajiwara et al., 2009).

Besides the Slitrk1 ECD, Sema3a expression is reduced in the Slitrk1 KO PFC of mice in the search for deregulated neurite development-controlling proteins (Figure 1C *NA neurite growth*; Hatayama et al., 2022). In the LC culture, Sema3a proteins inhibited neurite growth in wild-type LC neurons, but not in Slitrk1 KO-derived LC neurons (Hatayama et al., 2022), indicating that Sema3a also acts as a suppressive signal for NA neuron development and that the cell-autonomous functions of Slitrk1 involves mediating Sema3a signals (Figure 1C *NA neurite growth*). Thus, there are two possible contact-points between Sema3a signaling and Slitrk1 functionality. One is Slitrk1-mediated enhancement of Sema3a expression, and the other is Slitrk1-mediated Sema3a signaling facilitation.

The molecular basis for Sema3a signaling facilitation has been hypothesized that Slitrk1 deprives the Sema3a/Nrp1/L1CAM complex of L1CAM and affects the signaling efficacy of Sema3a (Figure 1C *Endocytosis*). This is because L1CAM, a Slitrk1-binding protein, serves as an NRP1 (neuropilin1, a Sema3a receptor)-associated signal transducing transmembrane protein (Castellani et al., 2000; Bechara et al., 2008), and L1CAM increases Sema3a receptor endocytosis (Castellani et al., 2004). Furthermore, because Sema3a acts as both a receptor and ligand in the bidirectional regulation of Sema3a signaling (Jongbloets and Pasterkamp, 2014), the reduction of Sema3a expression in the Slitrk1 KO PFC could be affected in the reverse direction. Although this hypothesis needs further validation, the functional linkages among Slitrk1, L1CAM, and Sema3a signaling seem to uncover a novel molecular function of SLITRK family proteins.

Consequently, both secreted Slitrk1 ECD and Sema3a are thought to be suppressive of NA fiber growth downstream of Slitrk1 in the PFC. Both cell-autonomous (actions of Slitrk1 proteins in LC neurons) and non-cell-autonomous (actions of Slitrk1 proteins in PFC neurons) are presumed to explain this biological function.

## 8. De-regulated neonatal monoaminergic signaling as a possible mechanism for neuropsychiatric disorders

During postnatal cortical development, the NA content transiently increases at the neonatal stage (NA surge; Figure 2A; mouse, P5–P7; rat, P0–P3; Levitt and Moore, 1979). During an NA surge, NA neurites extend rapidly in the PFC. Tangential NA fibers increases in the surface region (prospective layer I), where Slitrk1 is strongly expressed (Figures 2B,C). Such studies on Slitrk1 raised a possibility of the NA surge being the causal factor for some psychiatric disorders. Supporting this idea, many pharmacological or genetic studies have already shown the neuroplastic role of NA signaling in some neural circuits (Saboory et al., 2020).

### 8.1. α2 adrenergic receptor agonists

α2 adrenergic presynaptic receptors mediate the negative feedback of NA not only as autoreceptors (by suppressing NA release) but also as heteroreceptors (suppressing the release of serotonin, acetylcholine, and glutamate; Langer, 2015). α2 adrenergic receptor agonists have been used for the treatment of hypertension, attention-deficit/hyperactivity disorder, Tourette’s syndrome, various pain and panic disorders, symptoms of opioid/benzodiazepine/alcohol withdrawal, cigarette craving, and as adjuncts for sedation (Giovannitti et al., 2015; Langer, 2015; Fernandez et al., 2018).

Clonidine is an α2 adrenergic agonist that can enter the central nervous system. In rats, neonatal clonidine treatment reduces adult NA turnover and affects brain function (reviewed in Mirmiran et al., 1988). Specifically, daily subcutaneous injection of clonidine into rat neonates (P0–P21; twice a day, subcutaneous, 100 μg/kg) causes super-sensitivity to NA in hippocampal CA1 cells, and clonidine causes long-lasting plasticity as a result of low seizure susceptibility (Gorter et al., 1990). Clonidine administration during P8–P21 (P8, 8 μg/kg; P9, 25 μg/kg, twice a day, subcutaneous, 100 μg/kg) reduced rapid eye movement sleep during P8–P21 and increased locomotor activity at the adult stage (>P70; Mirmiran et al., 1983). In mice, clonidine treatment during the P1–P22 period (daily, subcutaneous, 35 μg/kg) resulted in a temporal delay in the appearance of developmental markers (surface righting, cliff aversion, rooting, pinna detachment, startle response, eye twitch, eye opening), reduced exploratory locomotor activity at P16, and impaired short-term memory in the novel object recognition task at P22 (Calvino-Núñez and Domínguez-del-Toro, 2014).

### 8.2. β2 adrenergic receptor agonists

Rats treated with a β2 adrenergic receptor agonist terbutaline during P2–5 (daily 10 mg/kg, s.c.) showed impaired development of the somatosensory cortex, hippocampus, and cerebellum (Rhodes et al., 2004), higher activity in an open field at P35, and altered acoustic startle responses at P42 in a sex-dependent manner (Zerrate et al., 2007). In humans, exposure to terbutaline during the third trimester for >2 days is associated with an increased risk of autism (Croen et al., 2011). In clinical terms, β2-adrenergic receptor agonists are used in bronchodilators and tocolytics. The adverse effects of β2-adrenergic receptor agonists *in utero* exposure include increases in autism spectrum disorders, psychiatric disorders, and poor cognitive, motor, and school performance-related functions, as well as changes in blood pressure (Witter et al., 2009). As mechanisms underlying the neurobehavioral signs in rodents, microglial activation associated with innate neuroinflammatory pathways has been proposed (Zerrate et al., 2007; Stowell et al., 2019; Sugama and Kakinuma, 2021).

### 8.3. Genetic studies

Some studies on attenuating neonatal NA signals are helpful in understanding its significance. Attenuation of α2A-adrenergic receptor expression in the neonatal rat brain reduces anxiety, acoustic startle response, and prepulse inhibition (Shishkina et al., 2004a,b). In KO mice of En2, a homedodomain-containing transcription factor-encoding gene expressed in the hindbrain, levels of serotonin, dopamine, and NA were dysregulated from P7 to P21 in En2-KO mice, although NA exhibited the greatest abnormalities, reduced ∼35% in the forebrain (Genestine et al., 2015). En2 KO mice exhibit depression-like behaviors and social approach deficits, both of which are rescued by desipramine (an NA reuptake inhibitor; Brielmaier et al., 2014). Inactivation of glucocorticoid receptors in the noradrenergic system influences anxiety- and depression-like behaviors in mice (Chmielarz et al., 2013).

## 9. Discussion

Overall, sufficient evidence seems to support the role of NA signaling in neuroplasticity. Although excessive neonatal NA, as found in Slitrk1 KO exerts some neuroplastic roles, whether excessive neonatal NA signaling has pathological meaning depends on future studies. To clarify this point, the following points are important. First, the mechanism for NA fiber development during the neonatal NA surge should be clarified. It is probable that NA fiber growth in the cerebral cortex occurs in conjunction with corticogenesis. However, we still may not have sufficient results for the control of neonatal NA fiber development, as suggested by the Slitrk1 KO study (Hatayama et al., 2022). Additional molecular cues or signaling mechanisms for modulating NA fibers in the cortex require further studies. Second, excessive neonatal NA signaling should be modeled in experimental animals. Adaptation of optogenetic or chemogenetic methods for the neonatal animals in a less stressful environment would facilitate the analysis. Moreover, given the modular organization of the LC output (Chandler et al., 2019), the selective modulation of the modules would be more informative. Third, the significance of α2 hetero-receptor-mediated plasticity can be addressed by combining appropriate monoamine sensors (Feng et al., 2019; Wan et al., 2021). The interaction between NA signaling and serotonin signaling likely occurs during the neonatal period, as suggested by the Slitrk1 KO phenotype. Serotonergic signaling is critical for neonatal development and neuroplasticity (Brummelte et al., 2017). Fourth, the effects of the neonatal NA surge on disease-associated neural circuits should be clarified. For OCRD, recent studies have proposed specific neural circuits that could mediate cognitive and affective processing defects in patients with OCD. Globally, cortico-striatothalamocortical circuits could be involved in OCD (Stein et al., 2019). Cortico-striatothalamocortical circuits comprise of parallel and partly segregated circuits involved in sensorimotor, cognitive, affective, and motivational processes. Specifically, the cortico-striatothalamocortical circuits include the dorsomedial PFC and ventromedial PFC, which are partly related to the mouse medial PFC. Recent chemogenetic and optogenetic studies have shown the medial PFC to be a hub for both the depression-like and anxiety-like behavior-associated neural circuits (Xia and Kheirbek, 2020; Biselli et al., 2021). Fifth, the entity of NA-mediated neuroplasticity can be clarified using appropriate models. These could be persistent changes in the neuronal circuit structure or altered epigenetic signatures that cause long-lasting changes in gene expression of functional molecules.

LC-NA dysfunction has been implicated in many disorders, including depression, anxiety, attention-deficit hyperactivity disorder, post-traumatic stress disorder, Alzheimer’s disease, and Parkinson’s disease (Poe et al., 2020). However, NA dysfunction is not a known specific cause of any symptomatology or disease process (Poe et al., 2020). Studies on SLITRK1 in this article may have provided us with genetic clues to consider the pathophysiological significance of NA surges and the neuroplastic role of NA.

## Author contributions

All authors listed have made a substantial, direct, and intellectual contribution to the work, and approved it for publication.

## Funding

Many ideas in this manuscript was obtained through studies supported by Kakenhi funds (22H02722, 20K06927, 19H03327, and 16K07057).

## Conflict of interest

The authors declare that the research was conducted in the absence of any commercial or financial relationships that could be construed as a potential conflict of interest.

## Publisher’s note

All claims expressed in this article are solely those of the authors and do not necessarily represent those of their affiliated organizations, or those of the publisher, the editors and the reviewers. Any product that may be evaluated in this article, or claim that may be made by its manufacturer, is not guaranteed or endorsed by the publisher.

## Footnotes

^1^https://bipex.broadinstitute.org/, https://schema.broadinstitute.org/, https://epi25.broadinstitute.org/

^2^ http://mousebrain.org/adolescent/

^3^ https://portal.brain-map.org/

^4^ https://services.healthtech.dtu.dk/service.php?SignalP
